# Enhanced Light Output Power on Near-Infrared Light-Emitting Diodes with TITO/Ag Multilayer Reflector

**DOI:** 10.3390/mi13050695

**Published:** 2022-04-28

**Authors:** Hyung-Joo Lee, In-Kyu Jang, Dae-Kwang Kim, Yu-Jung Cha, Sung Woon Cho

**Affiliations:** 1 CF Technology Division, AUK Corporation, Iksan 570-210, Jeonbuk, Korea; dieblood77@nate.com (H.-J.L.); jik8012@naver.com (I.-K.J.); kdkeorhkd@nate.com (D.-K.K.); 2 Department of Advanced Components and Materials Engineering, Sunchon National University, Sunchon 57922, Jeonnam, Korea; 3 Department of Energy Technology, Korea Institute of Energy Technology, Ujeong-ro, 72, Naju-si 58330, Jeonnam, Korea

**Keywords:** titanium–indium tin oxide (TITO), multilayer reflector, near-infrared, light-emitting diode (LED)

## Abstract

A titanium–indium tin oxide (TITO) multilayer reflector was investigated to improve the light efficiency of high-power, near-infrared, light-emitting diodes (NIR-LEDs). The TITO/Ag was fabricated by combining a patterned TITO and an omnidirectional reflector (ODR). For fabricating a high-power NIR-LED, the wafer bond process required the TITO reflective structure, which has patterns filled by AlAu contact metal, bonded directly to the Ag reflector deposited on the silicon wafer. Among Ag-based single- and multilayer reflectors, the TITO/Ag showed the highest reflectance (R = 96%), which was favorable for wafer-bonded high-power NIR-LEDs. Therefore, the TITO/Ag reflector enabled the production of wafer-bonded NIR-LED chips that exhibit superior output performance (190 mW) compared with conventional cases using a single Ag reflector.

## 1. Introduction

Near-infrared light-emitting diodes (NIR-LEDs) are actively being developed as optical input signal sources for health monitoring and smart mobility electronics, including heart rate sensors, time-of-flight sensors, small vehicles and flying drones [1,2]. To meet the demands of these applications, compact NIR-LEDs that can deliver higher power output at large injection currents are required. During the past few years, innovative approaches, such as multiple quantum wells (MQWs), distributed Bragg reflectors (DBRs), omnidirectional reflectors (ODRs) and current-spreading layers have been developed to improve the output power in NIR-LEDs [3,4,5,6,7].

Based on previous studies, the use of ODR in the wafer-bonding process can be an effective method of dramatically improving the light-emitting efficiency of LEDs [8]. Most of the photons emitted from the active region to the absorbing substrate are effectively reflected off the top surface or sidewall by the reflective function of the ODR. Generally, Ag single reflectors with superior reflectivity and excellent electrical conductivity have been applied to improve the light-emitting efficiency of LEDs. However, the easy thermal oxidation and thermal agglomeration of Ag single reflectors caused serious operational instability problems of LEDs. Therefore, some researchers have proposed a single reflector consisting of silver-based metal alloys with superior reflectivity and better thermal durability, such as AgCu, ITO/Ag (indium-doped SnO_2_) and AgIn, as new reflector candidates [9,10,11]. Meanwhile, some groups suggested a multilayer reflective structure for transparent insulator/Ag forms, such as Si_3_N_4_ and SiO_2_, which can improve reflectance performance by engineering optical transport paths [12]. Nevertheless, the use of resistive parts such as the metal alloy or insulating layer in reflector inevitably generates high power consumption and self-heating issues [13]. The reflective properties, electrical characteristics and thermal durability of ODRs can be effectively controlled by material selection and reflector architecture. Interestingly, transparent conductive oxide (TCO) materials, such as ITO and fluorine-doped SnO_2_ (FTO), can exhibit oxide-level transparent optical properties and excellent thermal stability, as well as metal-level low electrical resistance. Thus, the multilayer reflector of transparent conductor/Ag structural form using TCO layers may solve the aforementioned problems, such as low light-emitting efficiency, thermal instability and high device resistance. In this survey, as promising TCO/Ag multilayer reflectors, ITO/Ag and TITO/Ag (titanium–indium tin oxide/Ag) were explored to solve problems of conventional Ag single-layer and transparent insulator/Ag multilayer reflectors. In fact, the TITO/Ag multilayer reflector showed the highest reflectivity of 97% among several multilayer and conventional Ag single reflectors. Furthermore, NIR-LED chips using TITO/Ag exhibited the best output performance (190 mW) and low operation voltage characteristics, compared with cases using conventional Ag single-layer and explored multilayer reflectors.

## 2. Experimental Details

**Figure 1:**
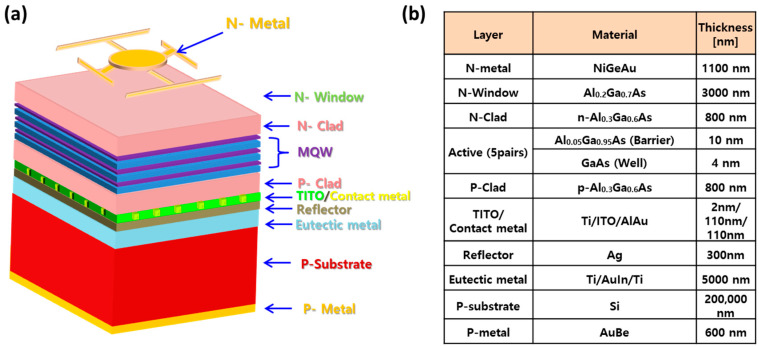
(**a**) Structural schematic and (**b**) composition information of wafer-bonded 850 nm near-infrared light-emitting diodes (NIR-LED).

The epitaxial wafers for an NIR-LED of 850 nm wavelength were prepared. Five pairs of gs, where 4 nm-thick GaAs (gallium arsenide) wells and 10 nm-thick Al_0.05_Ga_0.95_As barriers were alternatively stacked, were formed on the active region. The active region was located between the n- and p-type confinement layers, which are n- and p-Al_0.3_Ga_0.7_As materials, respectively. For the wafer bond process, n-GaInP and 5 μm-thick n-Al_0.2_Ga_0.8_As layers, grown sequentially on GaAs substrate, were employed as the current-spreading effect and etching-stop layer (ESL), respectively. Developed reflective structures with different optical thicknesses and repeated 10 µm-hole patterns were fabricated on n-Al_0.3_Ga_0.7_As layer. The Si_3_N_4_ (n = 2.0 at 850 nm) and ITO (n = 1.765 at 850 nm) had approximately 110 nm and 120 nm thicknesses, respectively. In the case of the TITO reflector layer, 2 nm-thick Ti was deposited on the top region of ITO layer using e-beam evaporator. There were approximately 80 patterns of 10 µm diameter in 340 µm × 340 µm NIR-LED chip. For bonding wafer to wafer, 400 nm-thick Ag and 5 μm-thick Ti/Au/In/Ti layers were used as the reflector and eutectic structures, respectively. After the wafer-bonding process, the GaAs substrate was selectively removed in the etchant with H_2_O_2_ and NH_3_ until the appearance of the GaInP layer. The GaInP ESL was eliminated in an HCl solution for 10 s. Water-bonded NIR-LED wafers were sequentially cleaned with acetone and methanol to remove organic contamination, which was followed by removing the surface oxidation of the n-Al_0.2_Ga_0.8_As top window (the front) and p-Si substrate (the back) in diluted HF etchant (HF:H_2_O = 10:1). After cleaning, the bonding pads were placed on the front and back using a combination of photolithography and selective etching. Note that Au/AuGeNi (1000 nm/100 nm) on the front was deposited using an electron beam evaporator, and Au/AuBe (500 nm/100 nm) was deposited on the back using a thermal evaporator. Finally, the reflective 850 nm NIR-LED chip using various reflectors, such as single- and multilayer reflectors, were fabricated. Figure 1a,b show the structural schematic and compositional information of the NIR-LED chip using TITO/Ag multilayer reflectors, respectively.

## 3. Results and Discussion

**Figure 2:**
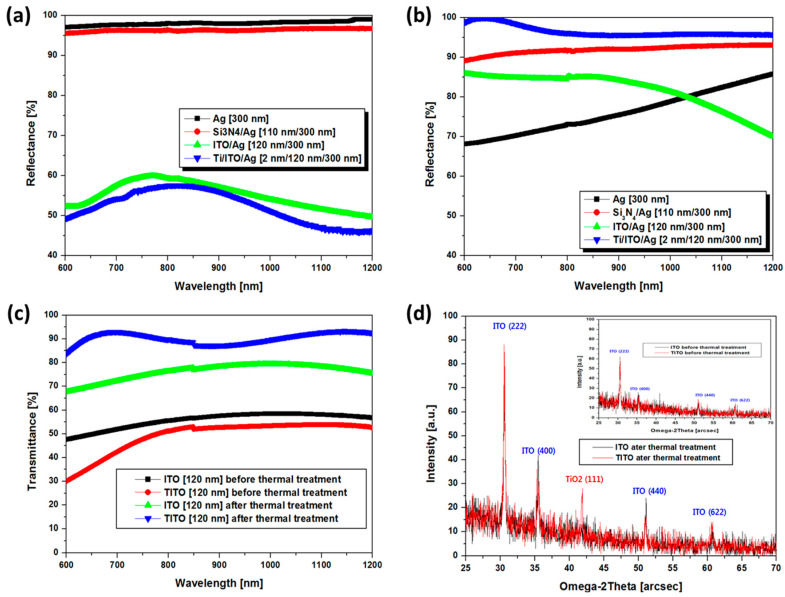
(**a**,**b**) Reflectance performances of Ag-based single-layer reflector (Ag) and multilayer reflectors (Si_3_N_4_/Ag, ITO/Ag, and TITO/Ag) before and after thermal treatment. (**c**) Optical transmittance and (**d**) X-ray diffraction (XRD) data of ITO and TITO before and after thermal treatment.

As shown in Figure 2, the optical reflectivity of various reflectors before (pristine state) and after thermal treatment (at 450 °C for 3 min) were evaluated via UV-Vis-NIR spectrometer (Waltham, MA, USA). As shown in Figure 2a, in the case of pristine states, both the single-layer (Ag) and transparent insulator/Ag multilayer (Si_3_N_4_/Ag) reflectors exhibit a high reflectance (R > 95%) over a wide spectral region of NIR light. On the other hand, TCO/Ag multilayer reflectors (ITO/Ag and TITO/Ag) show inferior reflection performance (R = ~56% at 850 nm), contrary to our expectation, which is disadvantageous for high-efficiency NIR-LED manufacturing. This is because pure ITO grown at room temperature has low crystallinity and low optical transparency due to a high disorder of metal cations and oxygen anions. Meanwhile, Figure 2b shows the reflectance performance of various reflectors annealed at 450 °C for 3 min. The Ag single reflector experience the significant degradation of optical reflectivity from 98% to 72% due to low thermal durability by thermal aggregation and oxidation. On the other hand, the Si_3_N_4_/Ag multilayer reflector showed a relatively insensitive degradation response to heat treatment though including an identical Ag layer. It means that the Ag layer covered by the insulating and the conductive layer in multilayer reflectors exhibits better thermal durability. In addition, the optical transparency of TCO layers (ITO and TITO) was improved after low-temperature thermal treatment, which induced significant improvement for the optical reflectivity of TCO/Ag multilayer reflectors (ITO/Ag and TITO/Ag). As shown in Figure 2c, the optical transparencies of the ITO and TITO layers after thermal treatment were approximately 75 and 85%. This is because the crystal quality of the TCO layers was dramatically improved due to the enhanced crystal quality by rearrangement of the metal and oxygen networks (Figure 2d). Especially, after thermal treatment, the TITO layer revealed higher transparency and better crystallinity, compared with the case of the ITO layer. It means that the thin-Ti layer plays a role of crystallization catalyst activating crystal growth of the ITO layer during thermal treatments [14]. It is already known that the Ti layer with high oxidation power is one of the good crystallization seeds for various transparent conductive oxides and semiconducting oxides [15,16]. During the thermal treatment, the metallic thin-Ti layer in TITO also was transformed into a transparent TiO_2_ layer due to thermal oxidation. After thermal treatment, the TITO/Ag multilayer reflector can exhibit the highest reflectance (R = 96%) which is most favorable for high-power NIR-LEDs due to the high transparency of TITO and the well-covered structure that prevents thermal damage to the Ag reflector.

**Figure 3:**
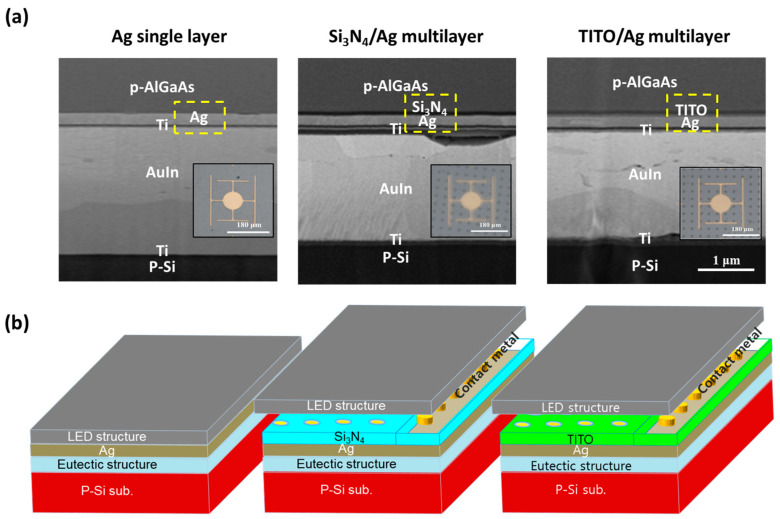
NIR-LED chips with Ag single, Si_3_N_4_/Ag and TITO/Ag multilayer reflectors; (**a**) cross-sectional SEM images and (**b**) structural schematics.

Figure 3 shows the cross-sectional scanning electron microscope (SEM) images and chip schematics of the 850 nm NIR-LEDs with Ag single reflector and multilayer reflectors (Si_3_N_4_/Ag and TITO/Ag). As shown in Figure 3a, for the fabrication of wafer-bonded NIR-LED with various reflectors, the epitaxial wafers with Ag-based single- and multilayer reflectors were bonded to a p-Si wafer through the wafer-bonding process. To obtain ohmic contact between the epitaxial layer (n-Al_0.3_Ga_0.7_As) and Ag reflector, the Si_3_N_4_, ITO, and TITO layers in multilayer reflectors were patterned with the 10 μm holes and those holes were filled with AlAu. All LEDs were manufactured with a reversed structure owing to the wafer-bonding process. As shown in Figure 3b, all reflectors were located between p-confinement at bottom of the epitaxial layer and the eutectic-structured top of the p-silicon substrate. All reflectors including Ag single- and multilayer reflectors were well prepared to effectively reflect upward photons emitting downward from the active region. The thickness of the Ag layer in all reflectors remains the same. In the case of LEDs using multilayer reflectors, the thicknesses of the Si_3_N_4_ film and the ITO film were approximately 100 nm. Additionally, all LEDs showed planar Lambertian distribution characteristics, regardless of the species of reflectors.

**Figure 4:**
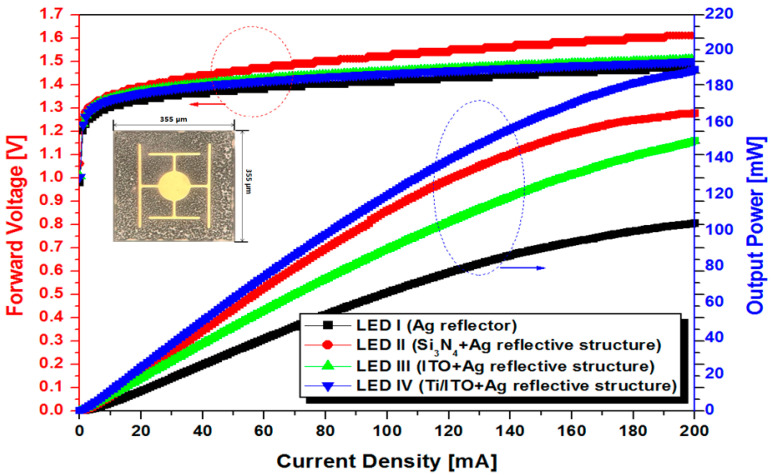
L-I-V curve for 850 nm LED chips using various reflectors: Ag (LED I), Si_3_N_4_ (LED II), ITO/Ag (LED III) and TITO/Ag (LED IV).

The 850 nm NIR-LED chips using LED I (Ag single reflector), LED II (Si_3_N_4_/Ag reflective reflector), LED III (ITO/Ag reflective structure), and LED IV (TITO/Ag reflective structure) were prepared to evaluate their performance. Figure 4 shows light output power–current (L-I) and current–voltage (I-V) characteristics of NIR-LED chips. Light emitted downwards from the active area is reflected upwards or laterally by the reflector, so the output power is greatly affected by the applied reflector. From the L-I curve in Figure 4, it was identified that there is a significant difference in the output power of the LED chips depending on the reflector. The NIR-LED chip using Ag single reflector revealed low output power (100 mW) at an injection current state of 200 mA. On the other hand, the introduction of multilayer reflectors in NIR-LEDs offered a significant improvement in output power. Especially, LED IV using TITO/Ag multilayer reflector with best reflectance performance showed the highest output power (190 mW), which increased by 90% compared with LED I, using Ag single reflector. This remarkable increase in output power of LED IV can be attributed to the excellent reflectivity of the TITO/Ag reflective structure, which is based on the high transparency and conductivity of the TITO film obtained with low heat treatment. Here, the output power results of the LEDs show a similar trend to the reflectance results of various reflectors. Meanwhile, the output power of LED II with multilayer reflector including insulating Si_3_N_4_ increased linearly up to 140 mA but saturated sharply above 140 mA. This is because the current diffusion effect decreases due to the increase in the injection current. Therefore, it can be expected that applying the insulating layer to the reflector is disadvantageous in reducing the operating power of the NIR-LED. In fact, from the I-V curve in Figure 4, it was identified that LED III using insulating Si_3_N_4_/Ag reflector required higher operating voltage condition, compared with the cases using conductive multilayer reflectors (ITO/Ag, and TITO/Ag). The result was attributed to the high conductivity of ITO film used in LED III and LED IV. These results clearly show that TITO/Ag multilayer reflectors are essential for improving the light-emitting efficiency and reducing the operating power of wafer-bonded 850 nm NIR-LEDs. In addition, the reliability of LED chips with Ag single- and multilayer reflectors was evaluated for 168 h under injection current of 150 mA. LED chips with Ag single reflector only showed significant degradation (25%) after 168 h. However, LED chips with Si_3_N_4_/Ag and TITO/Ag reflective structures could maintain their pristine emitting performance with less than 5% degradation properties. This is because the covering layers in multilayer reflectors such as Si_3_N_4_, ITO and TITO may interfere with thermal oxidation and thermal agglomeration of Ag layers in multilayer reflectors during long-term operation. It was confirmed that thermal aggregation problem of Ag reflector during long-term LED operation can be effectively improved by applying multilayer reflectors including Si_3_N_4_ and TITO films.

## 4. Conclusions

To develop high-power NIR-LEDs, various Ag-based multilayer reflectors, such as Ag single, Si_3_N_4_/Ag, ITO/Ag and TITO/Ag, have better reflectivity and thermal stability. Especially after thermal treatment, the TITO/Ag reflector showed the highest reflectivity of 97% among several multilayer and conventional Ag single reflectors. Meanwhile, the ITO/Ag and Si_3_N_4_/Ag reflectors revealed relatively low reflectivity of 85% and 92%, respectively. This is because the TITO layer with improved crystallinity through heat treatment can exhibit high transparency. Furthermore, LED chips with TITO/Ag reflectors can show linear output power trend and low operating voltage properties, compared with the case using Si_3_N_4_/Ag reflectors, due to the use of conductive TITO. As a result, we believe that TITO/Ag can be a desirable multilayer reflector for manufacturing high-power NIR-LED chips.
